# CD8α+ dendritic cells potentiate antitumor and immune activities against murine ovarian cancers

**DOI:** 10.1038/s41598-022-27303-7

**Published:** 2023-01-03

**Authors:** Shin-Wha Lee, Hyunah Lee, Kyung-Won Lee, Min-Je Kim, Sung Wan Kang, Young-Jae Lee, HyunSoo Kim, Yong-Man Kim

**Affiliations:** 1 Department of Obstetrics and Gynecology, Asan Medical Center, University of Ulsan College of Medicine, 88, Olympic-ro 43-gil, Songpa-gu, Seoul, 05505 Republic of Korea; 2 Pharmicell Co., Seoul, Republic of Korea; 3 Asan Institute for Life Sciences, Seoul, Republic of Korea; 4 Department of Obstetrics and Gynecology, GangNeung Asan Hospital, University of Ulsan College of Medicine, Gangneung, Republic of Korea

**Keywords:** Cancer, Immunology, Oncology

## Abstract

Dendritic cell (DC)-based immunotherapies have been shown to be a potential treatment option for various cancers; however, the exact strategies in ovarian cancer remain unknown. Here, we report the effectiveness of mouse CD8α+ DCs derived from bone marrow hematopoietic stem cells (BM-HSCs), equivalent to human CD141+ DCs, which have proven to be a highly superior subset. Mono-DCs from monocytes and stem-DCs from HSCs were characterized by CD11c+ CD80+ CD86+ and CD8α+ Clec9a+ expression, respectively. Despite a lower dose compared with Mono-DCs, mice treated with pulsed Stem-DCs showed a reduced amount of ascitic fluid and lower body weights compared with those of vehicle-treated mice. These mice treated with pulsed stem-DCs appeared to have fewer tumor implants, which were usually confined in the epithelium of tumor-invaded organs. All mice treated with DCs showed longer survival than the vehicle group, especially in the medium/high dose pulsed Stem-DC treatment groups. Moreover, the stem-DC-treated group demonstrated a low proportion of myeloid-derived suppressor cells and regulatory T cells, high interleukin-12 and interferon-γ levels, and accumulation of several tumor-infiltrating lymphocytes. Together, these results indicate that mouse CD8α+ DCs derived from BM-HSCs decrease tumor progression and enhance antitumor immune responses against murine ovarian cancer, suggesting that better DC vaccines can be used as an effective immunotherapy in EOC treatment. Further studies are necessary to develop potent DC vaccines using human CD141+ DCs.

## Introduction

More than 295,000 new cases of epithelial ovarian cancer (EOC) and approximately 184,000 EOC-related deaths were estimated worldwide by GLOBOCAN sources and methods in 2018^1^. EOC is the most fatal gynaecologic cancer, and unfortunately, there has been only a modest improvement in terms of overall survival in the last decade despite the introduction of promising cancer treatments. The standard treatment of EOC includes cytoreductive surgery and platinum- and taxane-based chemotherapy, and currently, the 5-year survival rate is 47% for all types of EOC. Bevacizumab is widely used as a frontline and second-line treatment for EOC^2^. In addition, based on the results of phase II/III trials, three poly (ADP-ribose) polymerase (PARP) inhibitors have been approved in the recurrent, platinum-sensitive setting as maintenance therapy after platinum-based therapy^3–5^. However, more than 70% of patients relapse after first-line treatment, even after achieving complete response, and the majority of platinum-sensitive patients eventually relapse. As a result, the average life expectancy does not exceed 1 year for platinum-resistant patients. This current EOC status presents an obvious unmet need for improving the survival and quality of life of patients.

Immunotherapy has recently received increasing attention and has demonstrated significant clinical benefit, leading to the approval of its use in several cancer types but not in EOC^6^. The representative novel immunotherapy is immune checkpoint blockade (ICB) targeting programmed cell death 1 (PD-1) and cytotoxic T-lymphocyte-associated protein-4 (CTLA-4), which has shown significant survival gain and durable responses. EOC is considered an immunoreactive cancer due to the presence of tumor-infiltrating lymphocytes (TILs)^7^. In addition, an immunoreactive molecular subtype characterized by an enrichment of genes and signaling pathways associated with immune cells was identified by The Cancer Genome Atlas Network^8^. However, in contrast to our expectation, the overall response rate (ORR) was only 15% in the first phase II study of nivolumab in recurrent EOC^9^ and only 8% in the phase II KEYNOTE-100 study of pembrolizumab, even though the ORR increased to 19% in patients with high PD-L1 expression^10^. These findings are considerably inferior to those in melanoma or lung cancer that display abundant intraepithelial TILs required for ICB responses. Therefore, to improve the effect of PD-1/PD-L1 monotherapy, combination therapy with CTLA-4 blockade has demonstrated favorable results in metastatic melanoma and lung cancer; however, serious toxicities related to the combined ICBs have resulted in the unmet need to develop new strategies for improved immunotherapy.

Dendritic cell (DC)-based antitumor vaccines have proven to be a safe therapeutic approach and have been used to boost antitumor immunity with promising results throughout recent decades^11–13^. DCs have been recognized as the most potent antigen-presenting cells (APCs), playing key regulatory roles in innate and adaptive immune responses. The effectiveness of DC vaccines remains a debate, mainly because of inconsistent clinical results. However, it is evident that these approaches potentiate strong tumor antigen-specific immune responses through the activation of cytotoxic T lymphocytes (CTLs) and increase cytolytic abilities by IFN-γ production. The limitations of ex vivo monocyte-derived DCs commonly used in current DC vaccines contribute to their lack of robustness. As a result, there has been a great effort to identify DC subsets with superior features to induce effective antitumor responses and apply them in therapeutic approaches. For this reason, human CD141+ DCs generated from CD34+ hematopoietic stem cells (HSCs), as conventional type 1 DCs (cDC1s), have been extensively studied in the development of DC-based therapies against cancer^14,15^; however, whether CD141+ DC therapies are effective in ovarian cancer remains unknown.

Prior to the investigation of the role of CD141+ DC vaccines in patients with EOC, the present study aimed to verify the effectiveness of mouse CD8α+ DCs derived from bone marrow (BM) HSCs in a murine model. Using a syngeneic and orthotopic mouse ovarian cancer model, we performed the most reasonable evaluation of mouse CD8α+ DCs, which correspond to human CD141+ DCs.

## Results

### DCs derived from stem cells present different characteristics compared with DCs derived from myeloid cells

**Figure 1:**
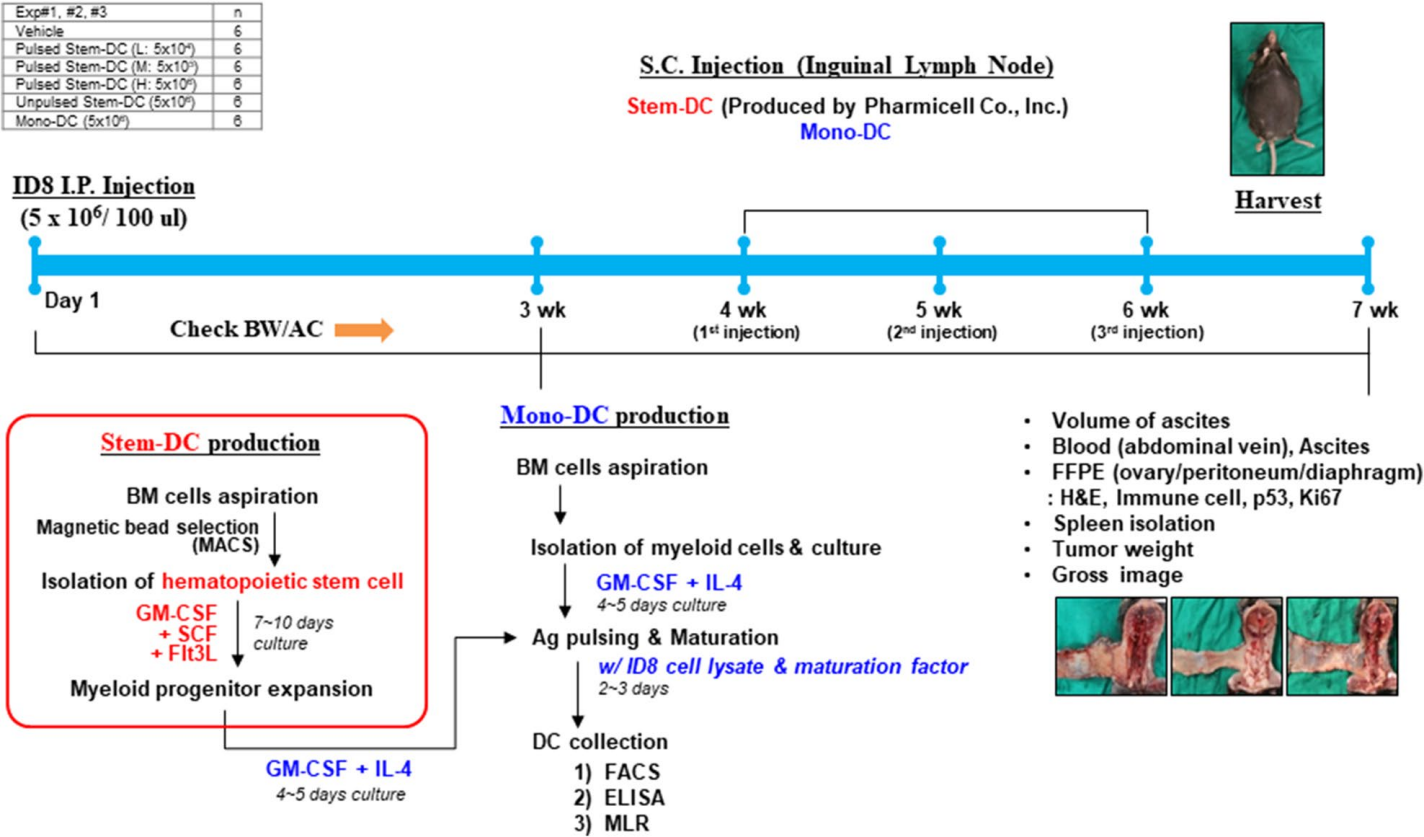
Workflow for the ovarian cancer orthotopic model using bone marrow-derived dendritic cells. We performed three independent experiments to evaluate the efficacy of mouse CD8α+ DCs originating from mouse BM stem cells, which correspond to human BDCA3+ (CD141+) DCs. An orthotopic model was established by intraperitoneally injecting ID8 mouse ovarian cancer cells. Six different treatments, including experimental groups of three doses of Ag-pulsed Stem-DCs, a comparative group of Ag-pulsed Mono-DCs, and two kinds of control groups treated with vehicle and unpulsed Stem-DCs, were analyzed. The immune responses before and after DC treatments and mouse reactions related to tumor formation and treatments were evaluated during and after DC treatments.

**Figure 2:**
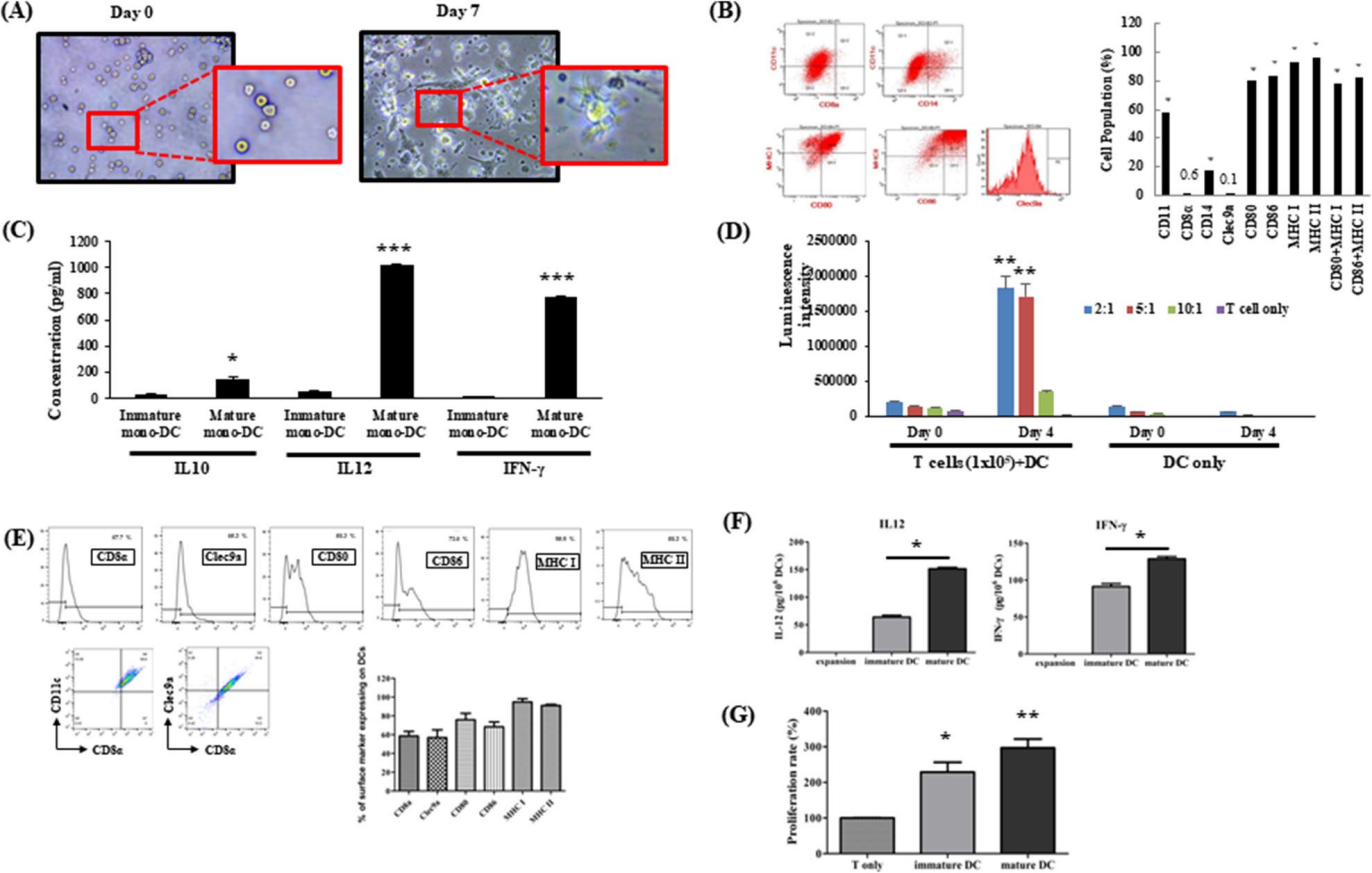
Characterization of mouse myeloid-DC (Mono-DC) and hematopoietic stem-DC (Stem-DC) derived from BM cells pulsed with tumor antigens of ovarian cancer. Mono-DC differentiation is shown from (**A–D**), and stem-DC differentiation is shown from (**E–G**). (**A**) Images of differentiated Mono-DCs at 7 days after BM cell cultures were treated with GM-CSF and IL-4 and pulsed with tumor cell lysates. (**B**) Flow cytometry analysis of differentiated Mono-DCs showing the expression of DC-specific cell surface markers and the absence of CD8α and Clec9a, which are intrinsic characteristics of mouse Stem-DCs. (**C**) Cytokine secretion by differentiated Mono-DCs with or without maturation factors was tested by ELISA at 24 h after stimulation (P = 0.0445, < 0.0001, and 0.0001, respectively). (**D**) T cell proliferation (MLR assay) with different amounts of Mono-DCs (2:1, DC 5 × 10^4^ cells + /− T cell 1 × 10^5^ cells; 5:1, DC 2 × 10^4^ cells + /− T cell 1 × 10^5^ cells; 10:1, DC 1 × 10^4^ cells + /− T cell 1 × 10^5^ cells; T cell only, T cell 1 × 10^5^ cells) (P = 0.0030 and P = 0.0044, respectively). (**E**) The typical phenotype of mouse Stem-DC (CD8α, Clec9a) was confirmed by FACS analysis. (**F**) IL-12 and IFN-γ secretion by differentiated stem-DCs with or without maturation factors was tested by ELISA immediately after culture (P = 0.0143 and P = 0.0286, respectively). (**G**) T cell proliferation (MLR assay) with stem-DCs in the presence or absence of maturation factors showed a significant difference compared with T cells only (P = 0.0012). The asterisks represent a statistically significant difference (*, P < 0.05; **, P < 0.01; ***, P < 0.001).

We evaluated the efficacy of Ag-pulsed stem-DCs originating from mouse BM stem cells using an orthotopic model by intraperitoneally injecting ID8 mouse ovarian cancer cells (Fig. 1). Mono-DCs from monocytes and stem-DCs from HSCs derived from mouse BM cells pulsed with tumor antigens of ovarian cancer demonstrated different characteristics (Fig. 2). Mono-DCs showed a distinct cellular morphology 7 days after BM cell cultures were treated with GM-CSF and IL-4 and pulsed with tumor cell lysates (Fig. 2A). Flow cytometry analysis of differentiated Mono-DCs revealed the expression of DC surface markers, including CD11c, CD80, CD86, MHC I, and MHC II. In addition, no expression of CD8α or Clec9a was observed, which are the intrinsic characteristics of mouse stem-DCs, indicating CTLs and myeloid lineage cells, respectively (Fig. 2B). Furthermore, immunostimulatory cytokines, such as IL-12 and IFN-γ, were secreted from mature Mono-DCs, and T cell proliferation was confirmed in proportion to the number of Mono-DCs (Fig. 2C,D). In contrast to Mono-DCs, the typical phenotype of mouse Stem-DCs induced by SCF and Flt3L was clearly confirmed as CD8α+ Clec9a+ cells (Fig. 2E). Similar to Mono-DCs, we identified not only the secretion of IL-12 and IFN-γ but also T cell proliferation induced by mature Stem-DCs (Fig. 2F,G).

### DC treatments affect tumor growth and ascites formation

**Figure 3:**
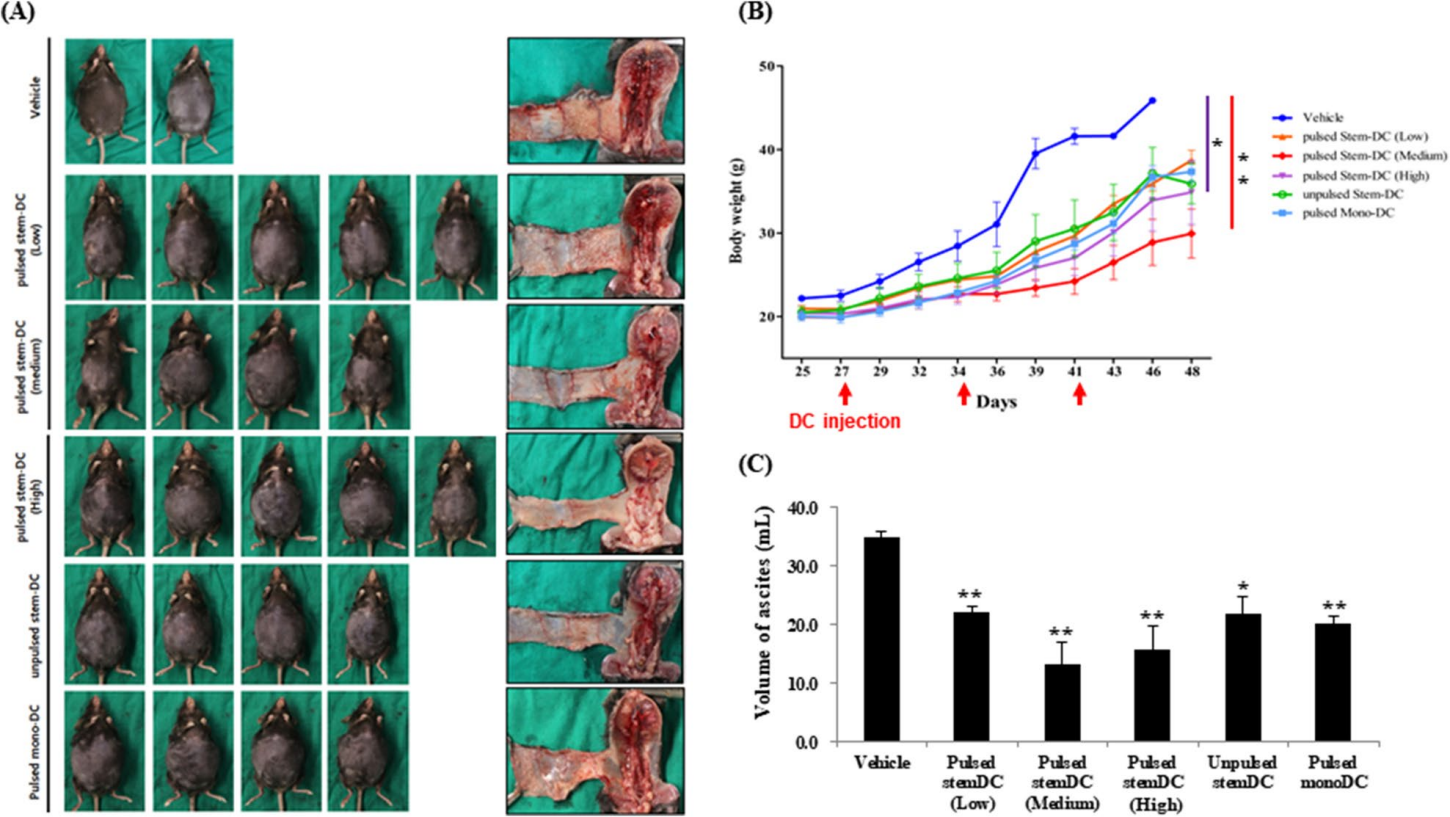
Effects of DC treatments on tumor growth and ascites formation in an orthotopic model bearing ovarian cancer. We harvested tissues from all mice treated with Stem-DCs, Mono-DCs, and vehicle between 8 and 9 weeks after injection with 5 × 10^6^ ID8 cells. (**A**) Images of the exterior appearance containing large ascites and multiple tumor-infiltrated internal organs after each DC treatment. In the vehicle group, only two mice were imaged because another four mice expired earlier than expected before the last DC injection because of tumor progression. (**B**) We analyzed body weight changes over time after DC injections. The data are presented as the mean and standard deviation. The high dose of pulsed Stem-DC and medium dose of pulsed Stem-DC groups showed lower body weights than the vehicle group (P = 0.0264 and P = 0.0092, respectively). (**C**) We carefully collected all ascites when harvesting. Compared with the vehicle group, all treated mice showed reduced ascites volumes (P = 0.0038, 0.0021, 0.0065, 0.0130, and 0.0012 in the order shown in the x-axis). The asterisks represent a statistically significant difference (*, P < 0.05; **, P < 0.01).

We harvested all mice treated with Stem-DCs, Mono-DCs, and the vehicle control between 7 and 8 weeks after the intraperitoneal injection of 5 × 10^6^ ID8 cells, although some mice in the vehicle control died earlier than we expected before the last DC injection because of tumor progression (Fig. 3). Most mice appeared to form ascites from 4 weeks after ID8 cell injection, which was confirmed by increasing abdominal circumferences. We observed significantly smaller body weights in the high- and medium-dose pulsed Stem-DC groups than in the vehicle group (P = 0.0323 and P = 0.0183, respectively). There were no differences in body weights according to the type of DC treatment among the DC-treated groups. When we compared the volume of ascitic fluid carefully collected at harvesting, all of the DC-treated mice showed a reduced volume of ascites.

### Tumor implants and tumor cell densities are decreased after DC treatments

**Figure 4:**
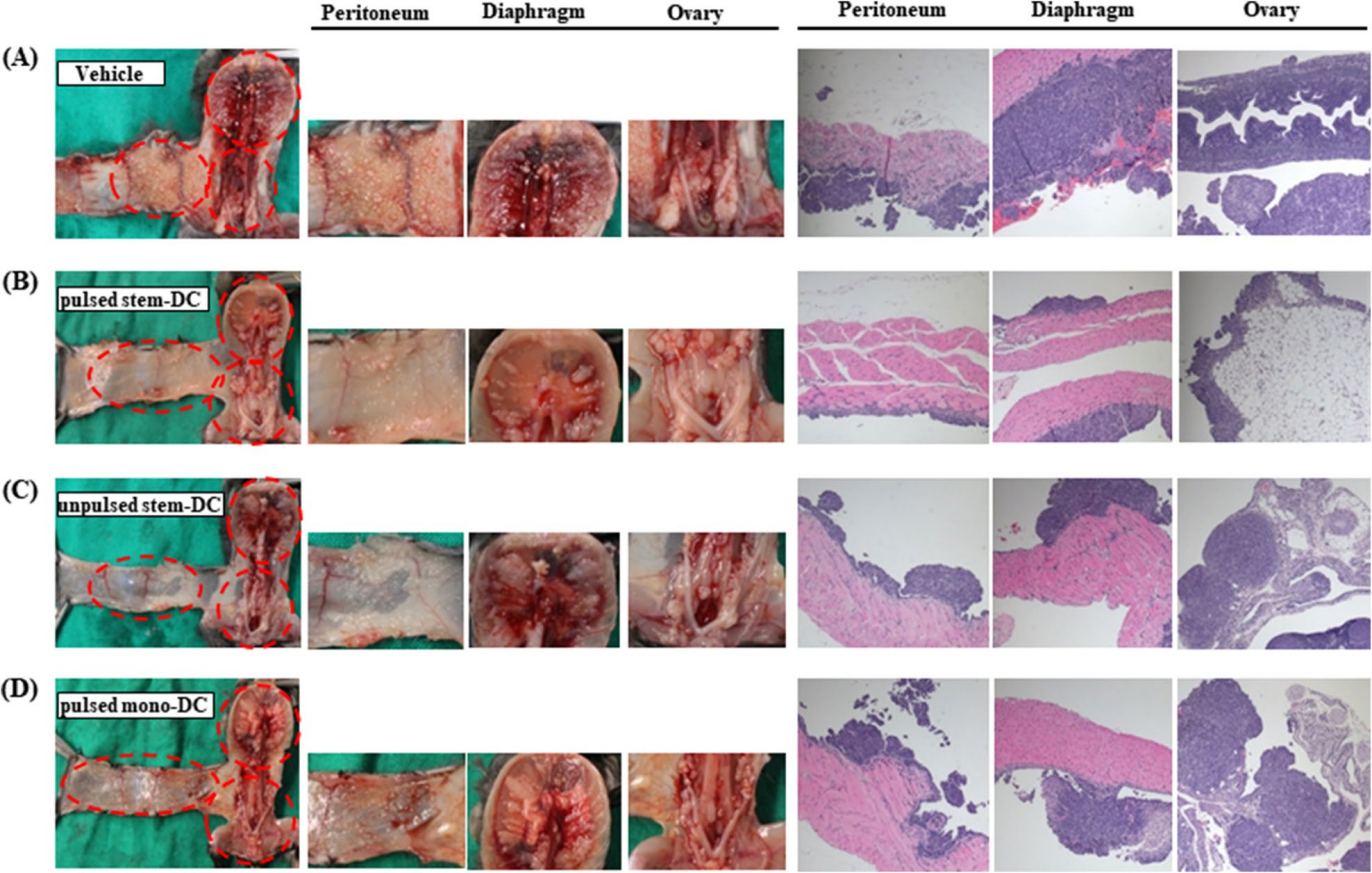
Different volumes of tumor implants according to DC treatments and histopathologic analysis. We intended to show representative images of multiple tumor implants on the peritoneum, diaphragm, and bilateral ovaries. (**A**) The vehicle group showed several tumor implants in the entire abdominal cavity and aggressive tumor invasion in H-E stained tissues. (**B**) Mice treated with pulsed stem-DCs (high dose) showed relatively fewer tumor implants, which were usually confined in the epithelium of each organ. (**C**) The unpulsed Stem-DC group, as a positive control, showed numerous miliary seeding nodules in the peritoneum and clear invasive tumor margins. (**D**) Additionally, numerous well-demarcated tumor implants were found in mice treated with pulsed Mono-DCs.

Although the number of tumor implants produced following ID8 cell injections appeared quite different according to the type of DC treatment, methods to quantitatively measure tumor volume in an orthotopic ovarian cancer model have not yet been developed. Therefore, we presented representative images showing multiple tumor implants on the peritoneum, diaphragm, and bilateral ovaries in each treatment group (Fig. 4). Many tumor implants with variable sizes in the entire peritoneal cavity and aggressive tumor invasion in H–E-stained sections were observed in the vehicle group. In contrast, mice treated with pulsed Stem-DCs showed relatively fewer tumor implants, which were usually confined in the epithelium of the ovaries, diaphragm and peritoneum. The unpulsed Stem-DC group, as a positive control, showed numerous miliary seeding nodules in the peritoneal cavity and comparatively clear tumor invasive margins microscopically and macroscopically. Additionally, numerous well-demarcated tumor implants were observed in mice treated with pulsed Mono-DCs. Representative H-E staining and p53 IHC assay results are shown in Fig. S1.

### DC treatments increase the survival of tumor-bearing mice

**Figure 5:**
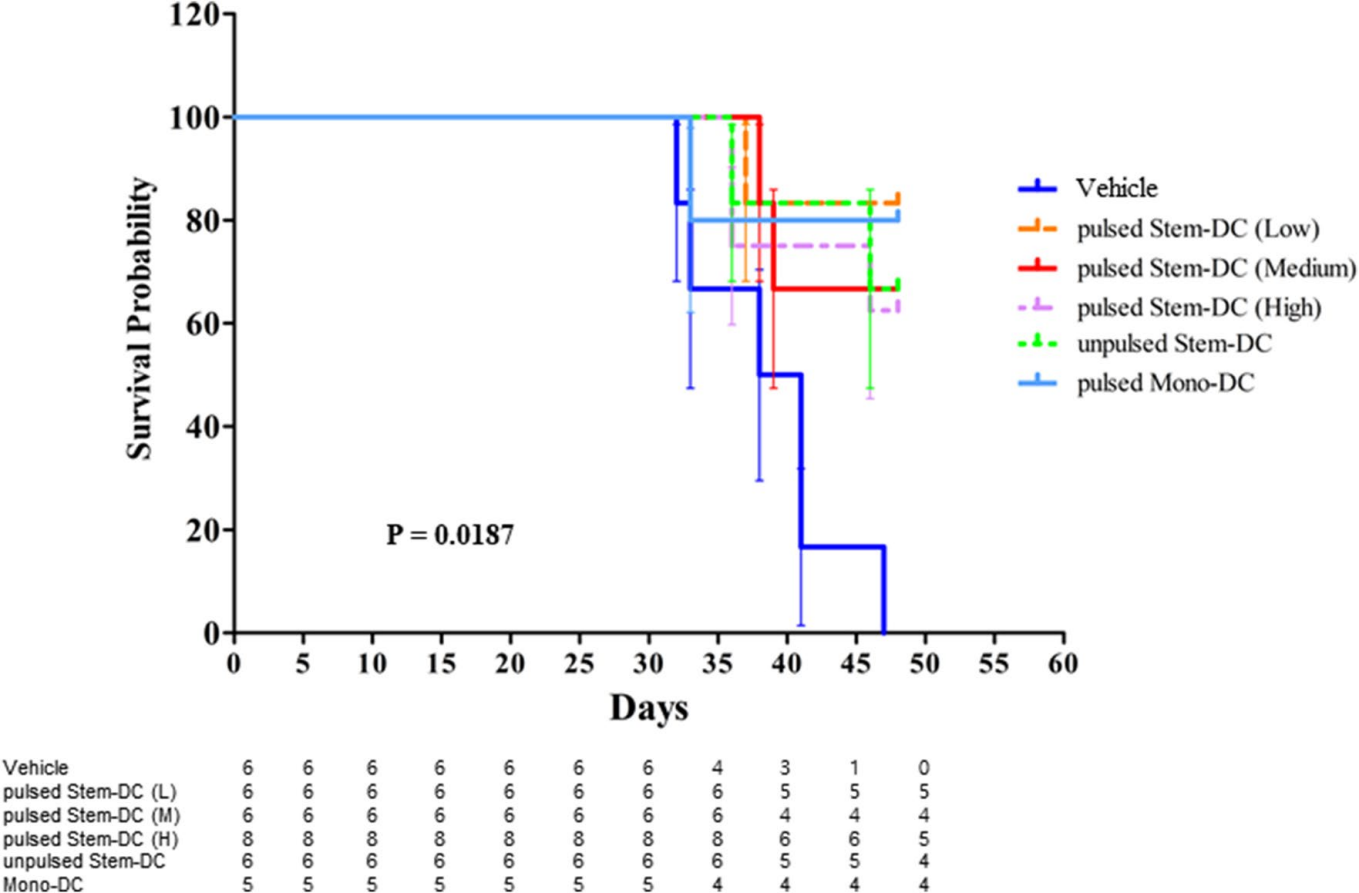
Comparative survival analysis according to DC treatments. We identified significant differences in survival in the vehicle group (P = 0.0187). All groups injected with DCs demonstrated longer survival than the vehicle group. For instance, there were significant differences in the medium- and high-pulsed Stem-DC groups compared with the vehicle group (P = 0.0323 and P = 0.0183, respectively). However, there was no difference according to the type of DC injected. This survival probability was analyzed by the log-rank (Mantel–Cox).

When survival was compared in six mouse groups, including the vehicle control, there was a significant difference in survival. (P = 0.0187) (Fig. 5). All mice injected with any type of DC demonstrated longer survival than the vehicle group. For instance, there were significant differences in the medium- and high-pulsed Stem-DC groups compared with the vehicle group (P = 0.0323 and P = 0.0183, respectively). There was no difference in comparisons between mice injected with DCs.

### Immune responses contribute to the significant effect of DC treatments

**Figure 6:**
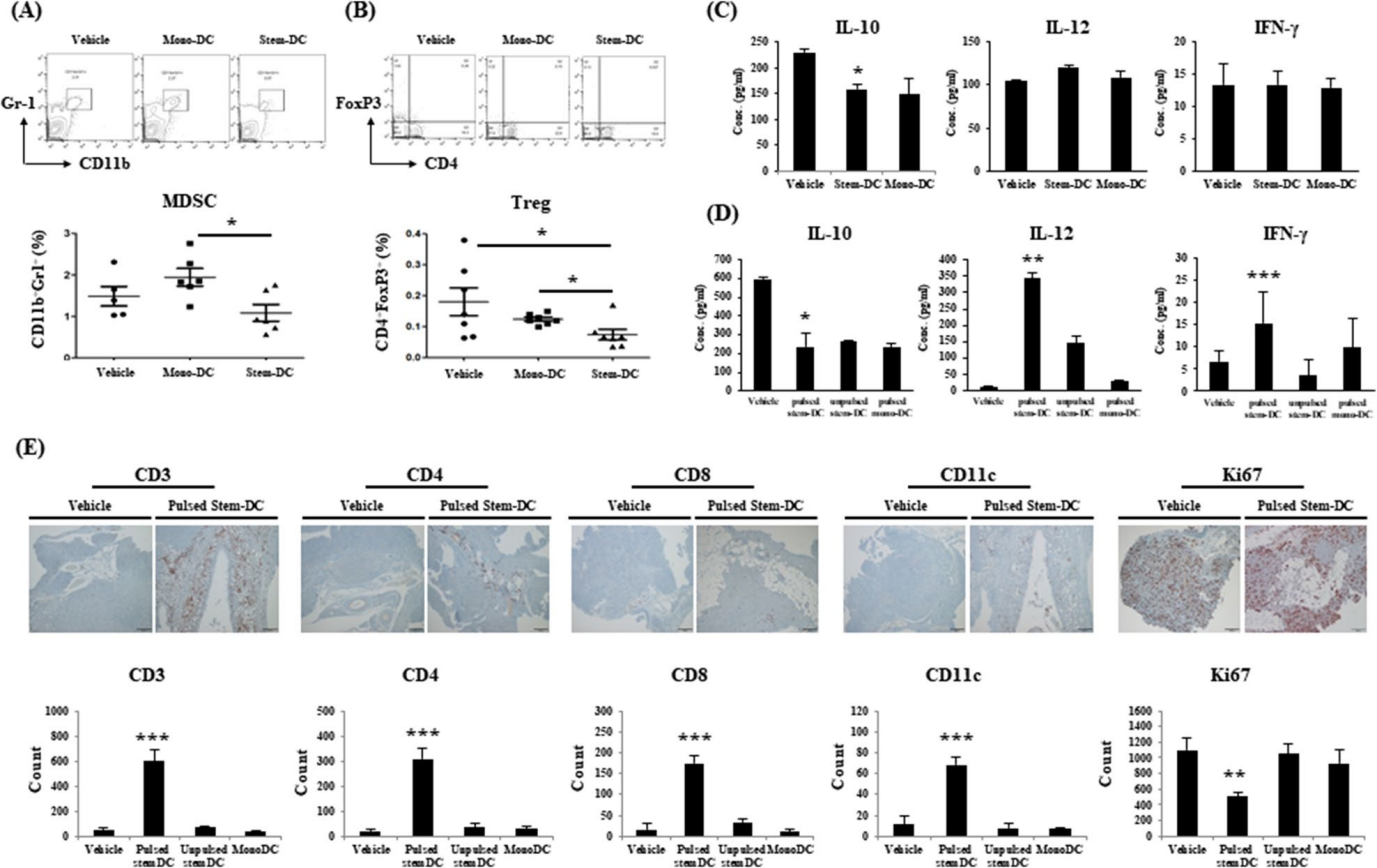
Immune responses in blood samples, ascites, and tumor tissues after DC treatments. We investigated immune reactions after DC treatments in the vehicle, Stem-DC (high dose), and Mono-DC groups. (**A**) In the FACS analysis, the proportion of MDSCs was lower in the serum of the pulsed Stem-DC group than in the serum of the Mono-DC group (P = 0.0141). (**B**) Similarly, in the FACS analysis, the proportion of regulatory T cells (Tregs) was significantly lower in the pulsed Stem-DC group than in the vehicle and Mono-DC groups (P = 0.0434 and P = 0.0132, respectively). (**C**) When we investigated the secretion of three cytokines in serum samples by ELISA, the level of immunosuppressive IL-10 was reduced in the pulsed Stem-DC group (P = 0.04199). In contrast, there were no differences in the immunostimulatory cytokines IL-12 and IFN-γ. (**D**) Unlike in the serum, ascites was significantly different in the pulsed Stem-DC group in terms of the secretion of cytokines, including IL-10, IL-12, and IFN-γ (P = 0.0347, 0.0073, and 0.0005, respectively). (**E**) We performed IHC staining to compare the number of representative immune cells. The expression levels of CD3, CD4, CD8, and CD11c were significantly higher in the pulsed Stem-DC group (P = 0.0008, 0.0006, < 0.0001, and < 0.0001, respectively). In contrast, the Ki67 level was significantly lower in the pulsed Stem-DC group (P = 0.0028). The asterisks represent a statistically significant difference (*, P < 0.05; **, P < 0.01; ***, P < 0.001).

To understand changes in the immune environment after DC treatments that are associated with tumor growth and survival, we investigated immune reactions in blood samples, ascites, and tumor tissues obtained after treatments with vehicle, high-dose Stem-DCs, and Mono-DCs (Fig. 6). The proportion of CD11b+ Gr1+ MDSCs was low in the serum samples of the pulsed Stem-DC group compared with the Mono-DC group (P = 0.0141), and the proportion of Tregs was significantly lower in the serum of the pulsed Stem-DC group compared with the vehicle and Mono-DC groups (P = 0.0434 and P = 0.0132, respectively). The immunosuppressive marker IL-10 was secreted at lower levels in the serum of the pulsed Stem-DC group (P = 0.04199), whereas immunostimulatory cytokines, including IL-12 and IFN-γ, showed no difference in the serum of mice according to the type of DC treatment. Unlike in serum samples, the levels of IL-10, IL-12, and IFN-γ were significantly different in the ascitic fluid of the pulsed Stem-DC group (P = 0.0347, 0.0073, and 0.0005, respectively). We did not observe different immune reactions in the spleen according to DC treatments (Fig. S2). Finally, we performed IHC staining to confirm the distribution of immune cells that are recruited in response to the tumor. The expression levels of CD3, CD4, CD8, and CD11c were significantly higher in the pulsed Stem-DC group (P = 0.0008, 0.0006, < 0.0001, and < 0.0001, respectively), and Ki67 expression was significantly lower in the pulsed Stem-DC group (P = 0.0028).

## Discussion

We evaluated the antitumor and immune responses of murine CD8α+ DCs. Among the characterized human DC subpopulations, cDC1s have emerged as a highly desirable tool for enhancing antitumor immunity, but existing approaches exploring cDC1s for antitumor immunotherapies have been limited to a few cancer types, such as melanoma, colon cancer, and lung cancer^16^. In this sense, our results are very encouraging and suggest a potentially effective new immunotherapy for ovarian cancer. The mouse CD8α+ Clec9a+ DCs (or stem-DCs) produced in this study demonstrated antitumor effects characterized not only by a reduced volume of ascites and number of tumor implants but also high levels of immunostimulatory cells and cytokines and low levels of immunosuppressive cytokines. Of course, all DC treatments have shown significant effects on the survival of tumor-bearing mice, but these antitumor and immune responses were significant even at relatively low therapeutic doses of Stem-DCs compared with Mono-DCs.

DC vaccines are the preferred approach to be implemented as a personalized cell-based therapy for cancer treatment, with more than 300 completed or ongoing registered clinical trials conducted to evaluate their application for boosting antitumor immunity^16^. Our authors have previously reported a phase I/II trial of a therapeutic DC vaccination with IL-2 as a consolidation therapy for ovarian cancer patients^17^. In that phase I/II study, 10 EOC patients with minimal residual disease after initial debulking and chemotherapy were treated with two subcutaneous doses of autologous monocyte-derived DCs pulsed with autologous tumor lysates and IL-2 to evaluate the safety and feasibility of this therapeutic strategy and characterize the antigen-specific immune alterations induced by this treatment. As a result, this DC vaccination was well tolerated and induced tumor-related immunity potentially associated with long-term clinical responses against EOC. In the three patients with long-term survival for 83, 81, and 38 months after DC vaccination without disease relapse, significant immune alterations were observed, including increased natural killer (NK) activity, IFN-γ-secreting T cells, immunostimulatory cytokine secretion, and reduced immunosuppressive factor secretion. Based on the DC studies of EOC published to date, many studies have focused on the selection of the antigen source. Hydrostatic pressure lysate preparation appears to be a crucial factor for DC vaccines to improve their efficacies, especially in ovarian cancer. Fucikova et al. reported that the high hydrostatic pressure used for preparing tumor lysates could induce immunogenic cell death, enhance DC uptake and cytokine release, and activate T cells in ovarian cancer^18^. In a recently reported phase I trial of a DC vaccine generated by the differentiation of autologous monocytes pulsed with oxidized autologous whole-tumor cell lysates in platinum-treated, immunotherapy-naïve, recurrent ovarian cancer patients, the DC vaccine was administered intranodally either alone, in combination with bevacizumab, or in combination with bevacizumab and low-dose intravenous cyclophosphamide until disease progression or vaccine exhaustion^19^. This vaccine induced T cell responses (increased IFN-γ production), and the antitumor immune response was associated with significantly prolonged survival, which was similar to our previous phase I trial^17^. Taken together, there are limited results of DC vaccinations for EOC. As a result, DC vaccines have not yet been approved as a standard treatment.

Although DC vaccination was introduced as a promising therapy after Sipuleucel-T approval in 2010, DC-based immunotherapies for the treatment of malignancies have generally shown limited clinical benefit. Among reported clinical trials with DC vaccines, the most common approach relies on the use of ex vivo differentiated DCs from leukapheresis-isolated monocytes cultured in the presence of GM-CSF and IL-4. However, although these DC vaccines are well tolerated, responses are achieved in less than 15% of patients^12,16^. These unsatisfactory outcomes are linked to immunosuppressive tumor microenvironments, such as checkpoint receptor signaling (PD-1/PD-L1, CTLA-4), and immune suppressive cells, such as Tregs and MDSCs. Moreover, in addition to TME characteristics, there are several limitations to the production of effective DC vaccines, including inadequate antigenic stimulation, suboptimal cell maturation^20^, lack of proliferative potential of DCs^21^, and expensive and complex manufacturing processes^22^. Therefore, the identification of a specific DC subset functionally skilled to achieve an effective antitumor immune response is essential.

Human DCs are represented by three major subsets in the aspects of diversified immune responses: CD141(BDCA3)+ Clec9a+ classical/myeloid DCs (cDC1), CD1c(BDCA1)+ classical/myeloid DCs (cDC2), and Clec4c(BDCA2)+ CD123+ plasmacytoid DCs (pDC)^23–25^. Although studies on CD141+ cDC1s began less than a decade ago, cDC1s have proven to be highly superior in terms of cross-presentation and to produce high levels of IL-12 and IL-15 to enhance the immunogenic function of NKs, natural killer T (NKT) cells, and CTLs^26,27^. However, cDC1s are very rare and account for < 0.03% of peripheral blood cells in humans^15^. Therefore, although there are no clinical studies with isolated cDC1 populations to date, CD141+ DCs are worth investigating for the development of potential therapeutic vaccines. There are several approaches to perform ex vivo differentiation of CD141+ DCs from human CD34+ progenitors using Flt3L, GM-CSF, SCF, thrombopoietin (TPO), IL-6, IL-3, and IL-4^15,16,28,29^. Moreover, preclinical studies have explored the use of cDC1s in some types of cancers using syngeneic mouse models and mouse CD8α+ DCs that are putative equivalents to human CD141+ DCs^30^. This present study is the first preclinical study to successfully show the possibilities of human cDC1 populations as an immunotherapy against ovarian cancer. We performed these experiments with well-differentiated mouse CD8α+ DCs supported by Flt3L, GM-CSF, SCF, and IL-4 in addition to pulsing by tumor cell lysates, which has turned out to be the most effective approach to produce CD141+ cDC1s. As a result, mice injected with pulsed Stem-DCs showed a smaller amount of ascites and tumor implants compared with those of the vehicle group, even at a relatively low injected DC dose (P < 0.05). This finding is supported by the absence of differences in pulsed Mono-DC injected mice. Furthermore, reduced tumor formation was associated with high expression of CD3, CD4, CD8, and CD11c in the IHC assay (all P < 0.001) and a reduced proportion of MDSCs and Tregs. In addition, low IL-10 and high IL-12 and IFN-γ levels were observed, which were significant in ascitic fluid. The immune response and tumor response after stem-DC treatments demonstrated the effect of murine CD8α+ DCs against ovarian cancer.

There are limitations to the present study that prevent the prediction of human CD141+ DCs as an effective EOC treatment. Most importantly, although our data indicate that CD8α+ DCs induce immune responses against ovarian cancer in the mouse model, the extrapolation to the human setting seems too far stretched. Therefore, further studies to investigate mouse CD8α+ DCs and human CD141+ DCs are necessary. Second, although we performed three independent experiments, there were only six mice in each treatment group. Last, because of the nature of the orthotopic model, tumor progression and immune monitoring after DC treatment were evaluated to a limited extent. It was feasible but not objective to compare the quantitative weight or size of tumors because our experimental model was intraperitoneal, so numerous small tumor implants inside the entire peritoneal cavity could not be counted exactly, and immune responses related to DC treatments were evaluated in restricted methods. Nevertheless, to the best of our knowledge, this is the first study to demonstrate the effectiveness of murine CD8α+ DCs in an ovarian cancer mouse model, suggesting that cDC1 vaccines represented by CD141+ Clec9a+ cells may be an effective immunotherapy in patients with ovarian cancer.

In conclusion, mouse CD8α+ DCs derived from BM-HSCs decrease tumor progression and enhance antitumor immunogenicity against murine syngeneic ovarian cancer. These findings suggest that a better understanding and development of DC vaccines can increase the effectiveness of ovarian cancer immunotherapy. Our results may provide useful information for the future development of more effective DC vaccines using human CD141+ DCs.

## Methods

### Study approval

All experimental protocols were approved by the Institutional Animal Care and Use Committee (IACUC) of the Asan Institute for Life Sciences. All methods were carried out in accordance with the guidelines and regulations of the IACUC of the Asan Institute for Life Sciences (protocol code 2017-12-082).

### Ex vivo generation of stem-DCs and mono-DCs

#### Isolation of BM-MNCs

Two different subtypes of DCs were generated from bone marrow mononuclear cells (BM-MNCs). BM-MNCs were obtained from the tibia and femur of C57BL/6 mice sacrificed by cervical dislocation. All experimental procedures were performed in accordance with the regulations of the Institutional Animal Care and Use Committee at the Asan Institute for Life Sciences.

#### Generation of stem-DCs

To generate hematopoietic stem cell-DCs (Stem-DCs), lineage-negative cells were selected from BM-MNCs by magnetic beads (MACS lineage depletion kit, Miltenyi Biotech, Germany) as HSCs. HSCs were seeded at a concentration of 1 × 10^6^ cells per well in 24-well plates and cultured in RPMI-1640 medium supplemented with 10% fetal bovine serum, granulocyte–macrophage colony-stimulating factor (GM-CSF, 100 ng/mL), stem cell factor (SCF, 50 ng/mL), and FMS-like tyrosine kinase 3 ligand (Flt3L, 50 ng/mL) (Peprotech, Rocky Hill, NJ, USA) for 7–10 days to promote their proliferation and differentiation into the monocyte lineage. Then, the cells were placed in culture media containing GM-CSF (1000 U/mL) and interleukin (IL)-4 (1000 U/mL) (Peprotech, USA) for 4–5 days to induce their differentiation into DCs.

### Generation of mono-DCs

Monocyte-DCs (Mono-DCs) were generated from the isolation of monocytes from BM-MNCs using a monocyte isolation kit (Miltenyi Biotech, Germany). Isolated monocytes were incubated for 4–5 days with GM-CSF (1000 U/mL) and IL-4 (1000 U/mL) (Peprotech, USA) to induce their differentiation into DCs.

#### Tumor antigen pulsing and DC maturation

For both stem-DCs and mono-DCs, ID8 ovarian cancer cell lysates as a tumor antigen were added to the culture media 2 days before the DC harvest. One day after tumor cell lysate pulsing, ginsenoside Rg3 (Sigma-Aldrich, St Louis, MO, USA) for stem-DCs and lipopolysaccharide (LPS) (Sigma-Aldrich, USA) for mono-DCs were added to the culture media as maturation factors for 24 h before the harvesting of DCs. Tumor cell lysates were prepared from cultured ID8 cells by a freeze–thaw process that was repeated six times in liquid nitrogen (− 180 °C) and an incubator (37 °C). Protein quantification of the tumor cell lysate was performed using centrifuged (1800 rpm, 10 min) supernatant by the Bradford method (Bio-Rad Laboratories, Hercules, CA, USA). Cultured cells were characterized as DCs by specific marker phenotyping, naïve T cell proliferation induced by DCs, and cytokine secretion.

### Formation and follow-up of the ovarian cancer orthotopic model and DC treatments

The orthotopic model of ID8 cancer cells was generated by injecting 5 × 10^6^ cells into the peritoneal cavity of C57BL/6 mice. Four weeks after cell injection, mice were categorized into six groups: the vehicle, low-dose pulsed Stem-DC, medium-dose pulsed Stem-DC, high-dose pulsed Stem-DC, pulsed Mono-DC, and unpulsed Stem-DC groups. Mice were subcutaneously injected with 5 × 10^6^ DCs for the high Stem-DC group, 5 × 10^4^ DCs for the low Stem-DC group, and 5 × 10^5^ DCs for the medium Stem-DC group around the inguinal lymph nodes. DCs were injected once a week for three weeks. We continued to assess the tumor status in mice by measuring body weight and abdominal circumference every two days after ID8 cell injection. One week after the last DC injection, mice were sacrificed, and tissues, such as the bilateral ovaries, peritoneum, diaphragm, and spleen, and several tumor implants were harvested and processed for histological analysis. At the time of harvest, we first carefully collected total ascitic fluid and blood samples from the abdominal vein.

### Immune response confirmation of generated DCs and DC treatments

#### Immunophenotypic analysis

Immunophenotypic analyses were performed to assess the characteristics of DCs and immune status after DC treatments by flow cytometry. The lymphocytes from mouse blood samples were prepared and purified by density gradient separation using lymphocyte separation medium (Mediatech, Manassas, VA, USA). Subsequently, the lymphocytes were stained with fluorescently labeled antibodies for 40 min at 4 °C. The following antibodies from eBioscience (San Diego, CA, USA) were used to confirm the characteristics of cultured DCs: anti-CD11c (11-0114-82), anti-CD80 (11-0801-81) for mouse DCs, anti-CD8α (17-0081-81) for CTLs, anti-CD14 (17-0141-82) for monocytes, anti-Clec9a (46-5975-82) for myeloid lineage cells, anti-CD86 (17-0862-81), and anti-MHC Class I (12-5958-80) and anti-MHC Class II (12-5320-80) for APCs. In addition, to accomplish immune monitoring after DC treatments, anti-CD11b (Cat. ), anti-Gr1 (Cat.) for myeloid-derived suppressor cells (MDSCs), anti-CD4 (Cat. ), and anti-FoxP3 (Cat.) for regulatory T lymphocytes (Tregs) were used. Single cells were analyzed using a FACSCanto II (BD Biosciences, Franklin Lakes, NJ, USA), and data were analyzed using FlowJo software (TreeStar, Ashland, OR, USA).

### Measurement of cytokine levels

Cytokine secretion was evaluated to confirm the characteristics of DCs and immune status after DC treatments using enzyme-linked immunosorbent assays (ELISAs). After the differentiation of DCs, mouse blood samples were obtained and stored at − 70 °C until ELISA was performed using ELISA kits for IL-10, IL-12, and interferon (IFN)-γ (R&D Systems, Minneapolis, MN).

### Lymphocyte proliferation assay

To assess the function of cultured DCs using mixed lymphocyte reaction (MLR) assays, syngeneic mouse splenic lymphocyte proliferation stimulated by DCs was observed. Responder splenic lymphocytes were cultured with or without effector DCs for 96 h at 37 °C in a 5% CO_2_-conditioned humidified incubator. Effector DCs were deactivated by treatment with mitomycin-C. At the end of culture, the Cell Counting Kit 8 (CCK8, Dojindo Laboratories, Kumamoto, Japan) reagent was added to each well and incubated for 3 h at room temperature. Absorbance at 450 nm was measured with an ELISA reader as an indication of the degree of DC-induced lymphocyte proliferation compared with lymphocytes only.

#### Immunohistochemical (IHC) assay

The presence of immune cells was investigated by IHC assays to describe the differences in the immune status according to DC treatments. Freshly obtained tumor tissues were fixed with 10% neutral buffered formalin for 8 h to 24 h at room temperature. After fixation, the tissue samples were rinsed, dehydrated, and embedded in paraffin blocks. Then, 3 μm-thick serial sections from the formalin-fixed paraffin-embedded tumor samples of mice were mounted onto glass slides (Muto Pure Chemicals, Tokyo, Japan). The slides were robotically prepared with an automated preparation system (Benchmark XT, Ventana Medical Systems, Tucson, AZ, USA). Deparaffinization, epitope retrieval, and immunostaining were performed according to the manufacturer’s instructions. Epitope retrieval was performed using cell-conditioning solutions (Ventana Medical Systems) at 100 °C for 60 min. For immunostaining, the ultraView Universal DAB Detection Kit (Ventana Medical Systems) was used. Tumor sections were stained with anti-CD3 (ab16669), anti-CD4 (ab183685), and anti-CD8 (ab217344) from Abcam (Cambridge, MA, USA) and anti-CD11c (97585, Cell Signaling, Danvers, MA, USA) antibodies at 37 °C for 36 min. In addition to immune cell markers, we investigated tumor markers using anti-Ki67 (ab16667) and anti-p53 (ab131442) primary antibodies purchased from Abcam.

#### Analysis of IFN-γ producing cells using enzyme-linked immunospot (ELISPOT) assays

The ELISPOT assay was used to detect and count individual cells that secreted IFN-γ protein in vitro upon exposure to an antigen. ELISPOT IFN-γ assay kits were purchased from AID (Strassberg, Germany) and used according to the manufacturer’s instructions. Splenic lymphocytes from tumor-bearing mice with or without DC treatment were stimulated in vitro with tumor lysates on a precoated 96-well plate. The plate was incubated for 20 h at 37 °C with 5% CO_2_. After washing, a detection antibody was added to each well and incubated for 2 h at room temperature. The plate was incubated with an alkaline phosphatase conjugate and developed with a BCIP/NBT substrate solution. Visible spots were counted using an automated AID ELISPOT reader (AID, Strassberg, Germany) and the default program.

### Statistical analysis

The mean value ± standard deviation was determined from at least three samples from different mice. All experiments were performed three times with DCs produced at different times. To compare means between two groups, the data were analyzed using the Mann–Whitney U test and considered statistically significant when the P value was < 0.05. Multiple group comparisons were analyzed by one-way analysis of variance, followed by a Friedman test and corrected using Dunn’s multiple comparison test. Survival was analyzed by the log-rank (Mantel–Cox) test. All statistical analyses were performed using GraphPad Prism 5.0 software (GraphPad software, San Diego, CA, USA).

### Ethics approval and consent to participate

The animal study protocol was approved by the Institutional Animal Care and Use Committee (IACUC) of Asan Institute for Life Sciences, and all experiments were performed in accordance with the regulations of IACUC of Asan Institute for Life Sciences (protocol code 2017-12-082 approved on May 02, 2017). All methods are reported in accordance with ARRIVE guidelines (https://arriveguidelines.org) for the reporting of animal experiments under ethics approval and consent to participate.

## Supplementary Information

Supplementary Information.

## Data Availability

The datasets supporting the conclusions of this article are included within the article.
