# Supplementary material for: CD8α+ dendritic cells potentiate antitumor and immune activities against murine ovarian cancers
Source: Sci Rep. 2023 Jan 3;13:98. doi: 10.1038/s41598-022-27303-7 (PMC9810613; doi:10.1038/s41598-022-27303-7)
Supplement: Supplementary file 1 — Supplementary Information. [file 41598_2022_27303_MOESM1_ESM.docx]

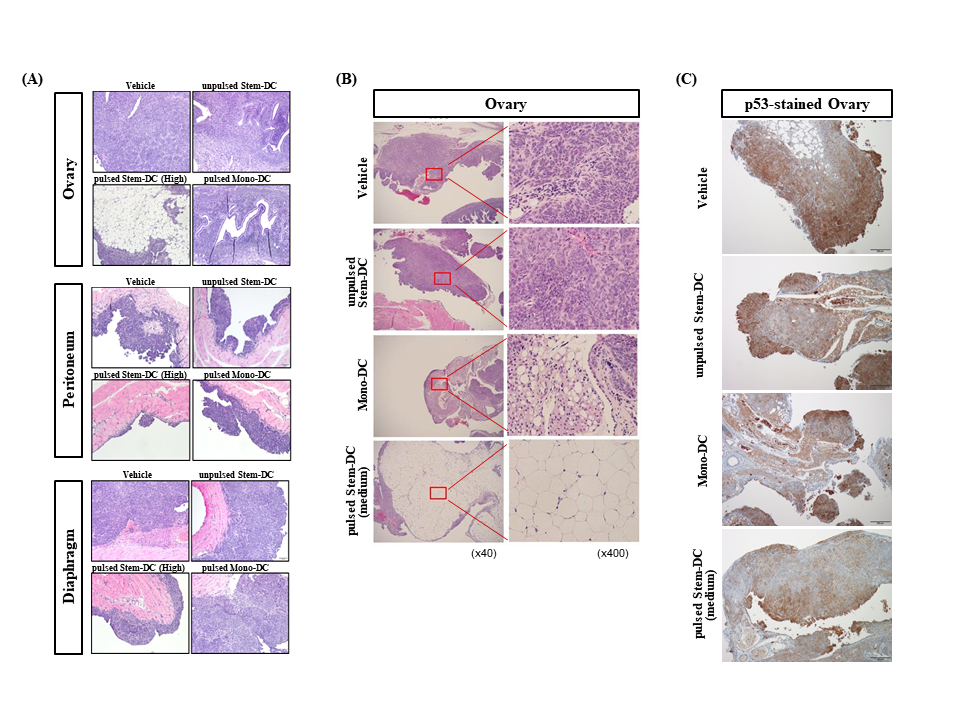


**Figure S1. Histopathologic analysis and immunohistochemical (IHC) assay of p53 expression.** (A) Remarkable aggressive tumor invasion was observed in the vehicle group regardless of the type of organ. However, pulsed Stem-DC treated mice demonstrated well-demarcated tumor implants that were mostly restricted to the epithelium. (B) H-E staining of ovaries at low and high magnifications. (C) IHC assay micrographs showing high p53 expression in tumor-bearing ovaries, which was similar among the different types of treatment.


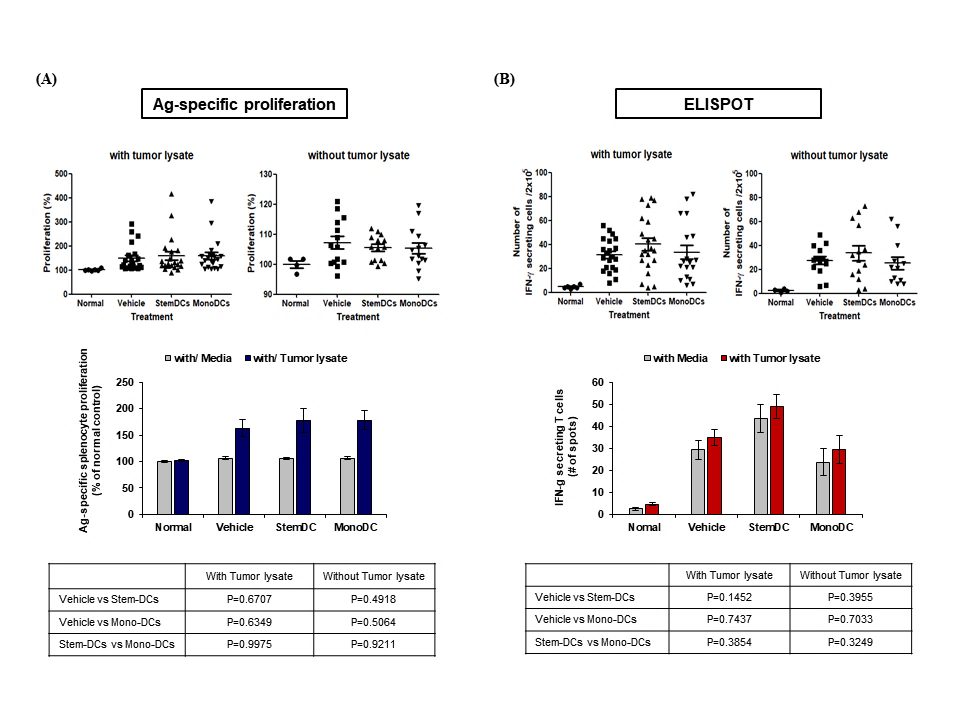


**Figure S2. Immune responses in the spleen after DC treatments.** We investigated immune reactions in the spleen after DC treatments in the vehicle, Stem-DC (high dose), and Mono-DC groups. (A) There were no differences in Ag-specific splenocyte proliferation regardless of the characteristics of the DCs generated. (B) There was also no difference in the ELISPOT results. IFN-γ secreting T cells were observed in similar amounts.
